# Dietary Patterns in Relation to General and Central Obesity among Adults in Southwest China

**DOI:** 10.3390/ijerph13111080

**Published:** 2016-11-03

**Authors:** Qiang Zhang, Xinguang Chen, Zhitao Liu, Deepthi S. Varma, Rong Wan, Qingqing Wan, Shiwen Zhao

**Affiliations:** 1 Department of Nutrition and Food Hygiene, Yunnan Center for Disease Control and Prevention, Kunming 650022, China; zhangqiang_cs@aliyun.com (Q.Z.); 15911621590@139.com (Z.L.); rongwan1963@126.com (R.W.); wanqingqing404@163.com (Q.W.); 2 Department of Epidemiology, University of Florida, Gainesville, FL 32610, USA; jimax.chen@ufl.edu (X.C.); dvarma@ufl.edu (D.S.V.)

**Keywords:** dietary patterns, factor analysis, obesity, adults, China

## Abstract

Dietary patterns represent a broader picture of food consumption, and are better correlated with a variety of health outcomes. However, few studies have been conducted to explore the associations between dietary patterns and obesity in Southwest China. Data from the 2010–2012 National Nutrition Survey in the province of Yunnan, Southwest China, were analyzed (*n* = 1604, aged 18–80 years). Dietary data were collected using the 24 h dietary recall over three consecutive days. Height, weight, and waist circumference were measured following standard methods. Exploratory factor analysis was used to identify dietary patterns. Logistic regression was used to explore the association between dietary patterns and obesity. Three distinct dietary patterns were identified, which were labeled as traditional, modern, and tuber according to their key components. With potential confounders adjusted, adults in the highest quartile of the modern pattern were at higher risk of general and central obesity (odds ratio (OR) 1.95, 95% confidence interval (CI) 1.15–3.48; OR 2.01, 95% CI 1.37–2.93). In contrast, adults in the highest quartile of the tuber pattern were at lower risk of general and central obesity (OR 0.34, 95% CI 0.15–0.61; OR 0.64, 95% CI 0.43–0.95) but at higher risk of underweight (OR 2.57, 95% CI 1.20–6.45). No significant association was found between the traditional pattern and obesity. Moreover, dietary pattern differences occurred due to the differences in socio-demographic characteristics. In conclusion, the modern dietary pattern was positively, and the tuber pattern negatively, associated with general and central obesity among adults in Southwest China.

## 1. Introduction

Obesity has become a major public health problem for both developed and developing countries [1,2]. Over the past twenty years, the prevalence of general and central obesity in Chinese adults has increased from 4.0% and 18.7% to 10.7% and 37.6%, respectively [3]. Increasing incidences of hypertension, type 2 diabetes, and other obesity related chronic non-communicable diseases (NCDs) have brought on a significant economic burden [4]. Obesity is a disorder that occurs as a result of interactions between genetic, environmental, and behavioral factors [5]. Rising prevalence of obesity can be best explained by the behavioral and environmental changes in modern times, especially in diet [6].

The traditional nutrient-effect approach has limitations to reveal the association between diet and obesity. In recent years, dietary pattern analysis has been widely used as an alternative method in nutritional epidemiology [7]. By assessing individual dietary exposure as a whole, dietary patterns are better associated with obesity. Moreover, the food combinations consumed reflect individual food preferences modulated by a mix of cultural, social, and economic determinants [8]. Studies in developed countries showed that consuming a diet high in fruit, vegetables, and whole grains and low in red meat and fast food was inversely associated with weight gain [9,10,11]. However, differences in environment and food culture limited the applicability of those findings to the Chinese population. The traditional Chinese diet is comparable to the Mediterranean diet and is considered to be healthy [12]. Nevertheless, a marked shift in Chinese diet has occurred with economic development and urbanization in the past decades [13]. In this process, traditional dietary habits have changed resulting in an increased incidence of nutritional diseases. Recent studies in China have assessed dietary patterns and obesity among children and adults living in different parts of China, but the results were not consistent [14,15,16]. Although the shift in diet is nationwide, they are occurring at markedly different rates across the country due to economic disparity [17]. More studies are needed to further understand the relation between dietary patterns and obesity in China.

As a less developed area, the prevalence of obesity has been relatively low in Southwest China [18,19]. However, recent studies from that region have shown that, while malnutrition still remains a serious problem, obesity is dramatically spreading [20,21]. This situation calls for further studies on obesity and dietary patterns in Southwest China. Using data from the latest wave of National Nutrition and Health Survey in the province of Yunnan, the purpose of the study was to identify and characterize dietary patterns, as well as assess their association with obesity in Southwest China.

## 2. Materials and Methods

### 2.1. Subjects

The analysis presented in the article was based on a subsample of the latest wave of National Nutrition Survey (2010–2012) in Yunnan, one of the less developed provinces in Southwest China. Participants were recruited using a stratified cluster sampling method. Four rural counties and two urban districts at different levels of economic development were included. From each of them, three townships were randomly selected. Then, two villages were randomly selected from each township. In each village, thirty households were randomly selected. All members in the households were invited to take part in the study. The response rate was 96.0%. Altogether, 1724 participants aged 18 years or over completed all parts of the study. One hundred and twenty participants were excluded because of pregnancy (36 subjects), diet (14 subjects), serious illnesses (25 subjects), or implausible energy intake based on the Chinese context (<500 kcal/day, >4200 kcal/day for men and >4000 kcal for women, 45 subjects) [22]. The final analysis was conducted on 1604 participants.

This study was approved by the Institutional Review Board at China Center for Disease Control and Prevention (2013-018). Written consent was obtained from all the participants before the survey.

### 2.2. Dietary Intake Measurement

Trained interviewers from local health institutions visited the selected households to collect information on dietary intake using the 24 h dietary recall over three consecutive days (including two weekdays and one weekend day). Energy and nutrient intake were calculated using the data of dietary records in conjunction with the China Food Composition Table [23]. Nutrient Adequacy Ratio (NAR, %) was used to determine the adequacy of energy, protein, and seven other micronutrients (calcium, iron, zinc, vitamin A, thiamine, riboflavin and vitamin C) in diets. NAR for a given nutrient was the ratio of a participant’s intake to the Recommended Nutrient Intake (RNI) in the Dietary Guidelines for Chinese. Socio-demographic information, lifestyle, and physical activity of each participant were also recorded using pre-coded questionnaires.

### 2.3. Dietary Patterns

Dietary patterns were identified using a factor analysis (principal component) of data from 3 days dietary recall [24]. Initially, 23 food groups were included. Due to the low intake of some food groups, they were further merged into 19 food groups (rice, wheat, tubers, beans, vegetables, fruits, meat, organ meat, oil, fish, milk, eggs, cakes, nuts, poultry, fast food, other wheat, liquors, and beer). Mean intake (g/day) of each food group over 3 days was used as input value in the analysis. Factors were rotated with an orthogonal rotation to achieve a more simplistic structure with greater interpretability. The number of factors retained was determined by eigenvalue (>1.0), scree plot, factor interpretability, and the variance explained (>5%) by each factor. Based on the analysis, three factors were selected. Labeling of the factors was based on an interpretation of the pattern structures. Factor loadings, which present the correlation between food groups and factors, were calculated for each food group across the three factors. Food groups with absolute values >0.25 are presented as figures in bold. Pattern-specific scores were calculated and assigned to each participant.

### 2.4. Anthropometric Measures

Body weight was measured to the nearest 0.1 kg, without heavy clothes. Height was measured to the nearest 0.1 cm, without shoes. Body mass index (BMI) was calculated as weight (in kilograms) divided by the square of height (in meters). Waist circumference (WC) was measured to the nearest 0.1 cm at the level of the iliac crest while the participant was at normal respiration. For each participant, WC was measured twice and the average was recorded. All measures were conducted by trained local physicians using a standard protocol. The Chinese BMI and WC cut-off points proposed by the Working Group on Obesity in China (WGOC) were used to define underweight, general obesity, and central obesity. Those measurements are as follow: underweight: BMI < 18.5; general obesity: BMI ≥ 28.0; central obesity: WC ≥ 85 cm for men and ≥80 cm for women [25].

### 2.5. Definition of Other Variables

“Low income” was defined as annual per capita income less than 5000 Yuan (US$750). “Low education” was defined as primary school and below. Smoking status, alcohol intake, and physical activity were included as potential confounders for their influence on weight [26]. Smoking status was defined as current smokers and non-current smokers. Current smokers were people smoking tobacco products at the time of the survey, while non-current smokers were not [27]. Alcohol intake was defined as non-drinker, moderate drinker (≤2 times/week), or heavy drinker (>2 times/week) [28]. Physical activity level was measured by the metabolic equivalent of task (MET) in hours per week [29]. MET was calculated according to the work, leisure, commute, and housework performed by the participants over the last 12 months.

### 2.6. Statistical Analysis

Each of the three factor scores was divided into quartiles from low to high (Q1–Q4). Continuous variables (e.g., age) were presented as mean ± standard deviation (SD), while categorical variables (e.g., gender) were presented as a percentage (%). Chi-square tests and linear regression analysis were used to examine the trend in categorical and continuous variables across quartiles, respectively. Logistic regression models with potential confounders adjusted were used to explore the correlation of dietary patterns with obesity. All statistical analyses were performed with SAS 9.4 (SAS Institute, Cary, NC, USA). Two sided *p* < 0.05 was considered statistically significant.

## 3. Results

### 3.1. Physical Characteristics of Participants

Physical characteristics of participants are summarized in Table 1. Of 1604 participants, 41.4% (*n* = 664) were men and 58.6% (*n* = 940) were women. The mean age of participants was 46.1 (SD 12.6) years. Mean BMI and WC were 23.3 kg/m^2^ and 80.7 cm for men, and 23.7 kg/m^2^ and 78.2 cm for women.

### 3.2. Dietary Patterns

Table 2 shows the three major dietary patterns among the participants. These dietary patterns explained 23.6% of the variance in dietary intake. The first one, characterized by a high intake of wheat, cakes, and oil, was labeled as the “traditional pattern”. The second one, which was highly correlated with vegetables, milk, eggs, meat, wheat, beans, and fast food, was named as the “modern pattern”. The last one, which included a high intake of tubers, fruits, and cakes, was labeled as the “tuber pattern”. These three dietary patterns explained 9.2%, 7.5%, and 6.9% of the variance in food intake, respectively.

### 3.3. Dietary Patterns and Socio-Demographic Characteristics

Socio-demographic characteristics of the participants across the quartiles of the three dietary patterns are shown in Table 3. People who preferred a traditional dietary pattern were more likely to live in urban areas and had higher educational levels. However, people who preferred the modern dietary pattern were older, had higher educational levels and income, were more likely to be male, lived in urban areas, and had higher physical activity levels. People who preferred a tuber dietary pattern were more likely to be female, lived in rural areas, had lower educational levels, and had lower physical activity levels.

### 3.4. Dietary Patterns and Nutrients Adequacy

Nutrient adequacy ratio and intake of energy and eight other nutrients across quartiles of the three dietary patterns are shown in Table 4. As a whole, the intake of energy, protein, iron, zinc, and thiamine among the participants were adequate (a NAR of 1.0 or greater). However, the intakes of four other nutrients were inadequate (a NAR less than 1.0), especially for calcium and vitamin A (NARs of 0.4 and 0.6, respectively). Participants with higher scores for the traditional pattern had lower intakes of energy, protein, zinc, and vitamin A. Higher scores for the modern pattern were associated with all of the nutrient intakes. NARs of calcium and vitamin A for participants in the highest quartile of the modern pattern were 0.6 and 1.1, respectively. Higher scores for the tuber pattern were associated with all the nutrient intakes, except vitamin A. However, the NAR of calcium was only 0.4, even for participants in the highest quartile of the tuber pattern. The percent energy from fat increased with the scores of the three dietary patterns and exceeded 30% for the highest quartile of the traditional and modern pattern.

### 3.5. Dietary Patterns and Obesity

The prevalence of underweight, general, and central obesity across quartiles of the three dietary patterns is shown in Table 5. The mean prevalence of underweight, general, and central obesity in the participants was 5.7%, 11.6%, and 37.9%, respectively. General and central obesity were more prevalent among adults with higher scores for the modern pattern. However, people with higher scores for the tuber pattern had a lower prevalence of general obesity and a higher prevalence of underweight.

Table 6 shows the odds ratio (OR) and 95% confidence interval (CI) for underweight, obesity, and central obesity across quartiles of the three dietary patterns with potential confounders adjusted. After adjusting for age, sex, energy intake, physical activity, smoke, and drink, participants with higher scores for the modern pattern were at a higher risk of general and central obesity (OR = 1.95, CI = 1.15–3.48; OR = 2.01, CI = 1.37–2.93). In contrast, people with higher scores for the tuber pattern were at a lower risk of general and central obesity (OR = 0.34, CI = 0.15–0.61; OR = 0.64, CI = 0.43–0.95) and a higher risk of underweight (OR = 2.57, CI = 1.20–6.45). No such association was found across the quartiles of the traditional pattern.

## 4. Discussion

We derived three dietary patterns by using factor analysis among adults in Yunnan, Southwest China: the modern, traditional, and tuber patterns. Further analysis indicated that the modern pattern was positively associated with general and central obesity. In contrast, the tuber pattern was negatively correlated with general and central obesity. No significant association was found between the traditional pattern and obesity or underweight. These associations were independent of age, sex, energy intake, physical activity, and other potential confounders.

As many developing countries, China has also experienced an accelerating nutrition transition due to economic and social development in the past several decades, resulting in significant changes in the dietary habits of the individuals [30,31]. The three dietary patterns we identified in this study were typical and mostly consistent with several previous national representative studies from China [14,32,33]. The modern pattern has been identified as a risk factor for metabolic syndrome and obesity in different countries and ethnicities [34,35]. Although modern patterns identified in different studies were not exactly the same, most of them had higher intakes of milk, fast food, and red meat. In general, the modern patterns represented an energy dense diet. In the present study, the highest loading for modern pattern was vegetables, which would have been a protective factor for obesity. However, higher intakes of milk, meat, and fast food in this pattern increased the risk of obesity. Our finding that a vegetable-rich food pattern was related to obesity is consistent with another study from the province of Jiangsu, East China [36].

Two traditional Chinese dietary patterns have been reported in previous studies: the north and the south. A traditional north pattern, characterized by a high intake of wheat, was positively associated with obesity in adults and adolescents [32,33]. This might be due to the high carbohydrate and low micronutrient content in this dietary pattern [37,38]. However, a traditional south pattern, which highly correlated with intake of rice, vegetables, and meat, is usually regarded as a protective factor for weight gain [24]. Although Yunnan is located in the Southwest region of China, the traditional pattern reported in the present study from this province was closer to the traditional north pattern reported in previous studies. This finding deepened our understanding of the commonality and individuality in Chinese diets. This study also found that vitamin A was negatively associated with the traditional pattern due to the lower intake of animal-source foods. This result is consistent with previous studies that suggested a possible risk of micronutrient deficiency for people following a predominantly traditional dietary pattern [15,33]. The tuber pattern used to be common in rural China but has seldom been reported in recent years [39]. This could be because of the dramatic decrease in tuber consumption across China during the past several decades [40]. As a diet low in fat, the tuber pattern was inversely associated with obesity and positively associated with underweight.

In addition to nutrition status, the three dietary patterns also captured the socio-demographic characteristics of the participants. The modern pattern was correlated with a higher income and education, which coincided with previous findings that nutrition transition began in urban areas and affected high income households first [41]. People following the modern diet usually had a low level of physical activity and represented an unhealthy lifestyle of new affluence in urban areas. The traditional pattern was not significantly correlated with income and presented a dietary pattern that had not changed. The tuber pattern correlated with a lower income and education, which represented a dietary pattern in rural areas. This finding is consistent with studies in some developing countries [42].

There were several limitations to this study. First, the data we used were cross-sectional; therefore, causal relations could not be determined. Second, the 24 h dietary recall method is inadequate to evaluate the usual dietary intake, and recall bias cannot be eliminated. Finally, the data for analysis were only collected from six counties in Southwest China. Caution is needed to generalize these findings to other places within China.

## 5. Conclusions

Despite the above limitations, our study assessed the association between dietary patterns and obesity in Southwest China for the first time. We found that the modern dietary pattern was related to an increased risk of obesity, while the traditional dietary pattern was not. The tuber dietary pattern was found to be inversely associated with obesity, and was rarely reported in the literature. This dietary pattern suggested the dramatic increase in the risk of malnutrition and obesity among adults in less developed areas of China. Prospective studies are needed to further understand the relationship between dietary patterns and obesity in Southwest China.

## Figures and Tables

**Table 1:** Physical characteristics of the participants.

	Men	Women	All
*n* (%)	664 (41.4%)	940 (58.6%)	1604
Age (Years)	46.9 ± 12.7	45.6 ± 12.4	46.1 ± 12.6
BMI (kg/m^2^)	23.3 ± 3.3	23.7 ± 3.8	23.5 ± 3.6
WC (cm)	80.7 ± 10.3	78.2 ± 10.4	79.2 ± 10.4

Age, body mass index (BMI) and waist circumference (WC) are presented as mean ± standard deviation (SD).

**Table 2:** Factor loadings for three dietary patterns among adults in Southwest China.

Traditional		Modern		Tuber	
**Wheat**	**0.69**	**Vegetables**	**0.63**	**Tubers**	**0.59**
**Cakes**	**0.55**	**Milk**	**0.49**	**Fruits**	**0.48**
**Oil**	**0.41**	**Eggs**	**0.44**	**Cakes**	**0.26**
Beans	0.23	**Meat**	**0.39**	Other wheat	0.24
Vegetables	0.22	**Wheat**	**0.30**	Vegetables	0.21
Organ meat	0.20	**Beans**	**0.28**	Wheat	0.17
Nuts	0.11	**Fast food**	**0.26**	Beans	0.16
Liquor	0.14	Poultry	0.22	Poultry	0.13
Poultry	0.12	Beer	0.21	Nuts	0.11
Fish	0.11	Oil	0.19	Organ meat	0.04
Other wheat	−0.03	Cakes	0.14	Eggs	−0.01
Tubers	−0.06	Nuts	0.11	Meat	−0.02
Meat	−0.10	Fruits	0.07	Rice	−0.05
Fruits	−0.14	Fish	−0.02	Oil	−0.13
Beer	−0.15	Rice	−0.07	Fast food	−0.18
Eggs	−0.20	Organ meat	−0.14	Milk	−0.24
Fast food	−0.21	Liquor	−0.20	**Fish**	**−0.32**
Milk	−0.24	Other wheat	−0.21	**Beer**	**−0.37**
**Rice**	**−0.65**	**Tubers**	**−0.37**	**Liquor**	**−0.48**
**Variance explained (%)**	**9.2**		**7.5**		**6.9**

Food groups with absolute values greater than 0.25 are presented as figures in bold. Variance explained is expressed as a percentage.

**Table 3:** Socio-demographic characteristics across quartiles of dietary patterns among adults in Southwest China.

	Dietary Pattern Quartiles				*p* for Trend
	Q1	Q2	Q3	Q4	
*n*	401	401	401	401	
**Traditional**					
Age (years)	49.3 ± 14.1	46.4 ± 14.1	46.7 ± 13.1	49.3 ± 14.5	0.88
Male (%)	37.4	44.9	44.0	38.5	0.82
Urban (%)	14.2	28.2	39.6	42.5	<0.01
Low Income (%)	34.2	40.9	31.6	36.0	0.72
Low Education (%)	69.8	66.8	56.2	50.0	<0.01
METs (h/week)	174.5 ± 140.1	177.5 ± 140.9	181.4 ± 141.7	170.5 ± 138.7	0.55
**Modern**					
Age (years)	45.6 ± 14.5	46.6 ± 13.8	48.0 ± 13.0	51.5 ± 14.0	<0.01
Male (%)	36.7	38.2	42.6	47.4	<0.01
Urban (%)	11.5	26.4	30.2	56.4	<0.01
Low Income (%)	43.4	36.9	31.4	30.9	<0.01
Low Education (%)	76.3	66.1	56.9	43.6	<0.01
METs (h/week)	165.7 ± 139.6	170.7 ± 139.0	177.5 ± 140.3	190.0 ± 144.3	<0.01
**Tuber**					
Age (years)	46.6 ± 13.9	48.6 ± 14.0	48.4 ± 14.0	47.9 ± 14.2	0.22
Male (%)	53.9	43.4	35.7	31.9	<0.01
Urban (%)	38.9	39.7	27.4	19.0	<0.01
Low Income (%)	36.2	35.7	33.9	36.9	0.96
Low Education (%)	56.1	54.9	65.1	66.8	<0.01
METs (h/week)	200.2 ± 146.3	181.1 ± 141.3	164.1 ± 139.0	158.5 ± 136.6	<0.01

Continuous variables (age and metabolic equivalents of task (METs)) are presented as mean ± SD; categorical variables (male, urban, low income and low education) are presented as a percentage.

**Table 4:** Nutrient intakes and adequacy across quartiles of dietary patterns among adults in Southwest China.

		Dietary Pattern Quartiles				*p* for Trend
		Q1	Q2	Q3	Q4	
*n*		401	401	401	401	
**Traditional**						
Energy	(kcal/day)	2460 ± 535	2002 ± 598	1878 ± 606	2170 ± 678	<0.01
	NAR	1.3 ± 0.3	1.0 ± 0.3	0.9 ± 0.3	1.1 ± 0.3	<0.01
Fat (% Energy)	17.5	23.2	28.4	31.2		<0.01
Protein	(g/day)	84.5 ± 25.2	71.5 ± 24.5	65 ± 25.8	71.5 ± 28.5	<0.01
	NAR	1.3 ± 0.4	1.1 ± 0.4	1.0 ± 0.4	1.1 ± 0.4	<0.01
Ca	(mg/day)	336 ± 125.1	312 ± 127.6	280 ± 147.0	336 ± 189.8	0.64
	NAR	0.4 ± 0.2	0.4 ± 0.2	0.3 ± 0.2	0.4 ± 0.2	0.67
Fe	(mg/day)	23.5 ± 6.9	20.8 ± 8.3	22.4 ± 9.0	24.6 ± 9.9	0.35
	NAR	1.5 ± 0.5	1.3 ± 0.6	1.4 ± 0.6	1.5 ± 0.5	0.58
Zn	(mg/day)	15.6 ± 3.7	13 ± 4.5	12.8 ± 4.5	12.3 ± 5.3	<0.01
	NAR	1.3 ± 0.3	1.0 ± 0.4	1.0 ± 0.3	1.0 ± 0.3	<0.01
Vitamin A	(µg/day)	532 ± 477.1	446 ± 398.2	417 ± 343.6	388 ± 306.1	<0.01
	NAR	0.7 ± 0.5	0.6 ± 0.5	0.6 ± 0.5	0.5 ± 0.5	<0.01
Thiamine	(mg/day)	1.3 ± 0.3	1.0 ± 0.4	1.1 ± 0.4	1.2 ± 0.4	0.12
	NAR	0.9 ± 0.3	0.7 ± 0.4	0.8 ± 0.3	0.8 ± 0.3	0.06
Riboflavin	(mg/day)	0.8 ± 0.3	0.8 ± 0.3	0.7 ± 0.3	0.9 ± 0.3	0.35
	NAR	0.6 ± 0.2	0.5 ± 0.3	0.5 ± 0.3	0.6 ± 0.3	0.22
Vitamin C	(mg/day)	87 ± 49.3	84 ± 47.9	82 ± 52.3	92 ± 64.4	0.64
	NAR	0.9 ± 0.6	0.8 ± 0.6	0.8 ± 0.7	0.9 ± 0.6	0.53
**Modern**						
Energy	(kcal/day)	1925 ± 570	1971 ± 540	2190 ± 526	2415 ± 546	<0.01
	NAR	1.0 ± 0.3	1.0 ± 0.4	1.1 ± 0.3	1.2 ± 1.2	<0.01
Fat (% Energy)		20.5	23.5	25.8	30.4	<0.01
Protein	(g/day)	61.1 ± 21.2	66.3 ± 22.9	76.7 ± 21.3	88.4 ± 27.6	<0.01
	NAR	0.9 ± 0.3	1.0 ± 0.3	1.2 ± 0.3	1.4 ± 0.4	<0.01
Ca	(mg/day)	192 ± 75.9	256 ± 88.7	344 ± 103.2	472 ± 92.6	<0.01
	NAR	0.2 ± 0.1	0.3 ± 0.1	0.4 ± 0.1	0.6 ± 0.2	<0.01
Fe	(mg/day)	19.2 ± 6.9	20.96 ± 7.8	21.6 ± 7.9	29.6 ± 9.9	<0.01
	NAR	1.2 ± 0.4	1.3 ± 0.4	1.4 ± 0.5	1.9 ± 0.6	<0.01
Zn	(mg/day)	11.3 ± 3.9	12.3 ± 4.2	13.7 ± 4.1	16.1 ± 4.9	<0.01
	NAR	0.9 ± 0.3	1.0 ± 0.3	1.1 ± 0.3	1.3 ± 0.38	<0.01
Vitamin A	(μg/day)	230 ± 111.3	302 ± 156.8	453 ± 261.1	799 ± 322.5	<0.01
	NAR	0.3 ± 0.3	0.4 ± 0.3	0.6 ± 0.3	1.1 ± 1.0	<0.01
Thiamine	(mg/day)	1.0 ± 0.4	1.0 ± 0.4	1.1 ± 0.4	1.4 ± 0.5	<0.01
	NAR	0.7 ± 0.3	0.7 ± 0.2	0.8 ± 0.3	1.0 ± 0.4	<0.01
Riboflavin	(mg/day)	0.5 ± 0.2	0.7 ± 0.2	0.8 ± 0.3	1.1 ± 0.4	<0.01
	NAR	0.4 ± 0.2	0.5 ± 0.1	0.6 ± 0.2	0.8 ± 0.3	<0.01
Vitamin C	(mg/day)	75 ± 46.8	70 ± 46.5	80 ± 52.2	120 ± 68.3	<0.01
	NAR	0.8 ± 0.5	0.7 ± 0.4	0.8 ± 0.51	1.2 ± 0.7	<0.01
**Tuber**						
Energy	(kcal/day)	1919 ± 571	2055 ± 610	2195 ± 626	2332 ± 646	<0.01
	NAR	0.9 ± 0.3	1.0 ± 0.3	1.1 ± 0.3	1.2 ± 0.4	<0.01
Fat (% Energy)		23	25.2	26	26.1	<0.01
Protein	(g/day)	64.3 ± 26.1	66.9 ± 19.2	78.0 ± 26.0	83.2 ± 28.1	<0.01
	NAR	1.0 ± 0.4	1.0 ± 0.3	1.2 ± 0.3	1.3 ± 0.5	<0.01
Ca	(mg/day)	272 ± 161.9	320 ± 108.8	336 ± 116.7	336 ± 163.1	<0.01
	NAR	0.3 ± 0.2	0.4 ± 0.2	0.4 ± 0.2	0.4 ± 0.2	<0.01
Fe	(mg/day)	20.8 ± 6.6	20 ± 6.5	23.6 ± 7.8	26.8 ± 10.2	<0.01
	NAR	1.3 ± 0.5	1.3 ± 0.5	1.5 ± 0.5	1.7 ± 0.6	<0.01
Zn	(mg/day)	11.7 ± 4.1	11.8 ± 3.5	13.7 ± 4.1	16.2 ± 4.9	<0.01
	NAR	0.9 ± 0.3	1.0 ± 0.3	1.1 ± 0.3	1.3 ± 0.5	<0.01
Vitamin A	(μg/day)	399 ± 242.6	460 ± 236.2	463 ± 294.2	462 ± 302.6	0.1
	NAR	0.5 ± 0.4	0.6 ± 0.4	0.6 ± 0.5	0.6 ± 0.5	0.2
Thiamine	(mg/day)	1.0 ± 0.4	1.0 ± 0.4	1.1 ± 0.4	1.4 ± 0.5	<0.01
	NAR	0.7 ± 0.3	0.7 ± 0.3	0.8 ± 0.3	1.0 ± 0.4	<0.01
Riboflavin	(mg/day)	0.7 ± 0.3	0.7 ± 0.3	0.8 ± 0.3	1.0 ± 0.4	<0.01
	NAR	0.5 ± 0.2	0.5 ± 0.2	0.6 ± 0.3	0.7 ± 0.2	<0.01
Vitamin C	(mg/day)	69 ± 44.1	73 ± 41.5	93 ± 69.1	115 ± 71.4	<0.01
	NAR	0.7 ± 0.5	0.7 ± 0.5	0.9 ± 0.6	1.2 ± 0.6	<0.01

Continuous variables (nutrient intake and nutrient adequacy ratio (NAR)) are presented as mean ± SD; categorical variable (% energy from fat) is presented as a percentage.

**Table 5:** Prevalence of underweight, obesity, and central obesity across quartiles of dietary patterns among adults in Southwest China.

	Dietary Pattern Quartiles			
	Q1	Q2	Q3	Q4
*n*	401	401	401	401
**Traditional**				
Underweight % (*n*)	7.2 (29)	4.7 (19)	6.5 (26)	4.3 (17)
General obesity % (*n*)	9.7 (39)	13.7 (55)	12.4 (50)	10.5 (42)
Central obesity % (*n*)	38.7 (155)	34.9 (140)	39.6 (159)	38.5 (154)
**Modern**				
Underweight % (*n*)	6.2 (25)	4.5 (18)	6.0 (24)	6.0 (24)
General obesity % (*n*)	6.5 (26)	11.7 (47)	13.2 (53)	15.0 (60)
Central obesity % (*n*)	24.7 (99)	39.7 (159)	40.7 (163)	46.6 (187)
**Tuber**				
Underweight % (*n*)	3.0 (12)	5.5 (22)	6.5 (26)	7.7 (31)
General obesity % (*n*)	11.7 (47)	14.2 (57)	13.7 (55)	6.7 (27)
Central obesity % (*n*)	37.9 (152)	41.7 (167)	38.4 (154)	33.7 (135)

**Table 6:** Multivariate adjusted odds ratio for underweight, obesity, and central obesity across quartiles of dietary patterns among adults in Southwest China.

	Dietary Pattern Quartiles				*p* for Trend
	Q1	Q2	Q3	Q4	
*n*	401	401	401	401	
**Traditional**					
Underweight	1.0	0.60 (0.28, 1.29)	0.66 (0.31, 1.41)	0.65 (0.30, 1.40)	0.29
General obesity	1.0	1.73 (0.99, 3.02)	1.71 (0.97, 2.99)	1.0 (0.56, 1.81)	0.70
Central obesity	1.0	1.07 (0.74, 1.56)	1.36 (0.94, 1.98)	0.92 (0.063, 1.34)	0.77
**Modern**					
Underweight	1.0	0.57 (0.24, 1.35)	1.19 (0.56, 2.50)	1.31 (0.62, 2.74)	0.19
General obesity	1.0	1.66 (0.90, 3.07)	1.71 (0.93, 3.13)	1.95 (1.15, 3.48)	0.02
Central obesity	1.0	1.93 (1.32, 2.83)	1.69 (1.15, 2.47)	2.01 (1.37, 2.93)	<0.01
**Tuber**					
Underweight	1.0	1.94 (0.79, 4.77)	2.06 (0.83, 5.12)	2.57 (1.20, 6.45)	0.03
General obesity	1.0	1.13 (0.69, 1.86)	1.21 (0.73, 2.01)	0.34 (0.15, 0.61)	<0.01
Central obesity	1.0	1.02 (0.72, 1.44)	0.99 (0.68, 1.42)	0.64 (0.43, 0.95)	0.02

Adjusted for age, sex, energy intake, physical activity, smoking and alcohol intake. The values are shown as odds ratio (confidence interval).
